# Broadband MIR harvester using silicon nanostructures

**DOI:** 10.1038/s41598-019-42022-2

**Published:** 2019-04-09

**Authors:** Sara Magdi, Farah El-Diwany, Mohamed A. Swillam

**Affiliations:** 1 Department of Physics, School of Sciences and Engineering, American University in Cairo, AUC Avenue New Cairo, 11835 Cairo, Egypt; 2 Nanotechnology Program, School of Sciences and Engineering, American University in Cairo, AUC Avenue New Cairo, 11835 Cairo, Egypt

## Abstract

In this work, we propose an all-silicon-based super absorber in the mid infrared (MIR) spectral range. The presented structures are composed of n-doped silicon nanoparticles or nanowires embedded in intrinsic silicon. An intense absorption peak is observed and could be tuned across the MIR range. While nanoparticles give a single broad absorption peak, the nanowires structure shows a broadband absorption of more than 70% from λ = 5 to 13 µm reaching up to 99% at 7 µm. The absorption peak could be extended to more than 20 µm by increasing the length of the nanowire. Increasing the diameter of the nanoparticles gives higher absorption, reaching just above 90% efficiency at λ = 11 µm for a diameter of 1500 nm. Changing the geometrical parameters of each structure is thoroughly studied and analyzed to obtain highest absorption in MIR. The proposed structures are CMOS compatible, have small footprints and could be integrated for on-chip applications.

## Introduction

Thermal harvesting has become an important requirement for various applications^1^. The efficiency degradation and long term instability of solar cells due to over-heating of the semiconductor material caused by absorbing photons with energy higher than the band gap has urged the need for thermal photovoltaic devices^2^. Harnessing mid infrared (MIR) light has also become crucial for infrared imaging, bio-sensing and other medical applications^3^. Harvesting this thermal energy requires MIR broadband absorbers made from CMOS compatible materials that have small footprints and could be easily fabricated and integrated for on-chip applications^3–6^. Thus, non-metallic MIR absorbers, such as semiconductors, will undoubtedly open new directions for increasing light-matter interaction in this spectral range^3,7^. Another advantage for using semiconductors is the tunability of the carrier densities, which adjust the resonant wavelength over a large part of the MIR range^7–9^.

Semiconductor nanostructures have recently gained increased attention due to their ability to trap visible light for solar cell devices^3,10,11^. Therefore, using them to design a perfect absorber in the MIR range has become of great importance. Doped silicon nanostructures are very attractive materials because of their abundance, fabrication maturity, CMOS compatibility, low toxicity and low cost in addition to their resonance in the MIR range^7,8,12,13^.

**Figure 1:**
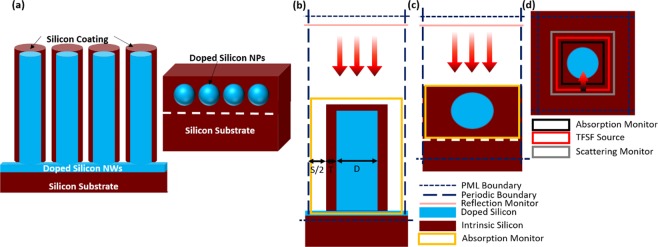
(**a**) 3D schematic of the proposed structures. (**b**,**c**) Simulation region for (**b**) NWs and (**c**) NPs structures. (**d**) Simulation region for calculating Mie efficiency for doped Silicon NPs in a Silicon environment.

In this work, we propose all-silicon simple structures containing n-doped silicon nanoparticles (NPs) or nanowires (NWs) embedded in intrinsic crystalline silicon. The 3D schematic of these structures is shown in Fig. 1(a). These designs provide broadband absorption without the need for multiple resonators or large footprint structures. Whereas a single broadband absorption peak is observed for periodic doped silicon NPs, a multimodal broadband absorption from 5 µm to 13 µm is achieved for periodic doped silicon NWs coated with intrinsic silicon. These structures provide excellent light trapping capabilities for MIR light, which significantly enhances light-matter interaction at this spectral range. The advantage of the proposed structure lies mainly in being an ultra-broad band absorber based completely on one silicon material that absorbs light in a huge band in the MIR spectrum and could be easily fabricated. Silicon nanowires fabrication is well established in literature and could be done using lithography free and low-cost techniques such as metal assisted chemical etching and excimer laser making this structure feasible in terms of less fabrication complexity and low cost^14–16^. In addition, the diameters of the nanostructures used in this work are as small as several nanometers and with a maximum of 1.9 µm. Thus, the proposed structure has small footprints compared to other structures with multiple resonators and large footprints^17^.

## Methods

**Figure 2:**
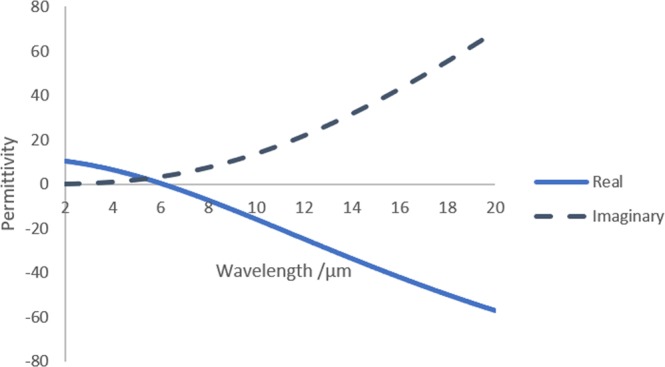
Complex permittivity of doped silicon versus wavelength for N_d_ of 1 × 10^20^ cm^−3^ which indicates plasma wavelength of value 1.7 µm.

The dispersion of the doped silicon was calculated using Drude model. The following equation was used for obtaining the permittivity ε(ω):

$${\rm{\varepsilon }}({\rm{\omega }})={\varepsilon }_{\infty }-\frac{{\omega }_{p}^{2}}{{\omega }^{2}+j\omega {\rm{\Gamma }}}$$

where ${\varepsilon }_{\infty }$ = 11.7 is the permittivity at ω = $\infty$, $\Gamma$ is the scattering rate ($\Gamma =q/{m}^{\ast }\mu$) where μ is the carrier mobility, ω_p_ is the plasma frequency and given by:

$${\omega }_{p}=\sqrt{\frac{{N}_{d}{q}^{2}}{{\varepsilon }_{o}{m}^{\ast }}}$$

where N_d_ is the doping concentration, q is the charge of electron, ${\varepsilon }_{o}$ is the permittivity of free space and ${m}^{\ast }\,$is the effective mass and equals 0.26 m_e_ where m_e_ is the free electron mass. The complex permittivity is shown in Fig. 2.

To study the proposed structures, finite difference time domain (FDTD) simulations are performed. Mie extinction, scattering and absorption cross sections are initially calculated for isolated NPs in a silicon environment using a total field scattered field (TFSF) source. The simulation region is shown in Fig. 1(d) and contains the TFSF source surrounding the nanostructure, as well as two monitors for calculating the absorbed and scattered light. The monitor inside the field source calculates the net power flowing into the particle (i.e. absorbed power), while the monitor outside the source calculates the net power scattered from the particle. In this simulation, the light is injected at one edge of the monitor and then subtracted at the other edge. Thus, the reflected and scattered light are subtracted from the incident light and the remaining is considered as absorbed light (it is trapped inside the nanoparticle). Absorption and scattering cross sections are then calculated using post scripting as absorbed and scattered power, respectively, normalized to the input source intensity. The extinction cross section is the sum of both the scattering and absorption cross sections. Mie efficiency is subsequently calculated as:

1 $${\rm{Mie}}\,{\rm{Efficiency}}=\frac{{\rm{Calculated}}\,{\rm{Cross}}\,{\rm{Section}}}{{\rm{Area}}\,{\rm{of}}\,{\rm{the}}\,{\rm{Particle}}}$$

The boundaries surrounding the nanostructure are perfectly matched layer (PML) to absorb all the power reaching it and to avoid boundaries’ reflections. The span between the boundaries is 4 µm to ensure that any reflections from the boundaries would not affect the calculations. For NWs, absorption and scattering cross sections are calculated in a silicon environment using the same technique implemented for the NPs. However, only 2D simulations are performed to calculate the cross sections for the NWs.

Doped silicon NPs and NWs are then added into a thin film silicon layer and shined with a plane wave source (λ = 4–20 μm). The absorption is measured using a 3D electrical field monitor coupled with a 3D refractive index monitor to differentiate between the absorption inside doped and intrinsic silicon. Using post scripting, sweeping over multiple parameters is conducted to obtain high and broadband absorption. The simulation region for this part is shown in Fig. 1(b,c), where the upper and lower boundaries are set to PML and the side boundaries are periodic. The two monitors enclose the silicon materials to measure the absorption inside them while the whole structure resides on a silicon substrate.

## Results and Discussions

### n-doped silicon NPs

**Figure 3:**
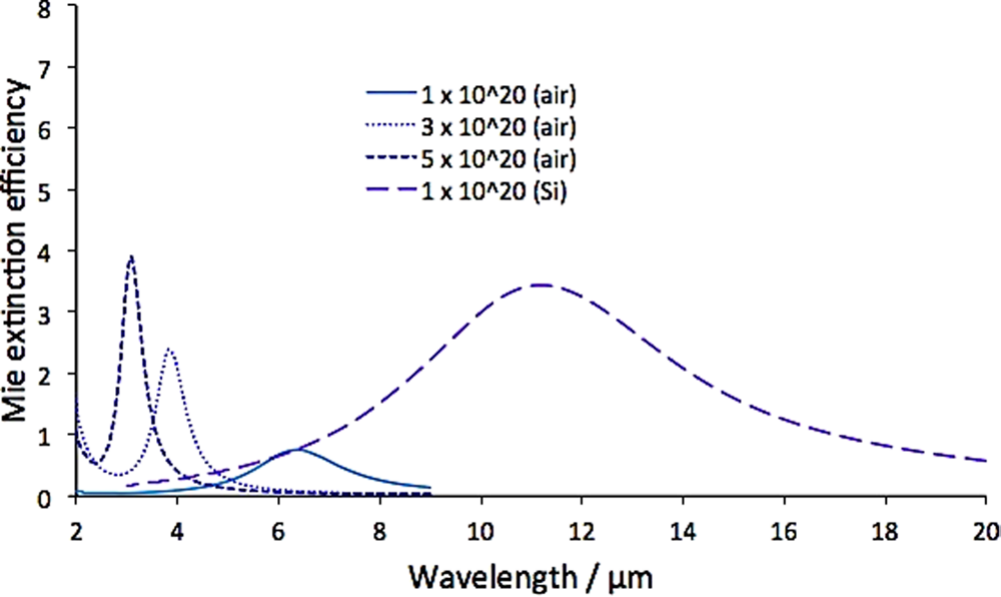
The relationship between the doping concentration and Mie extinction efficiency in air and silicon environment, using 500 nm-sized nanoparticles with a period of 2000 nm.

To calculate the Mie efficiency, the simulated structure is composed of a doped silicon NP of diameter range 500–1900 nm, surrounded by either air or crystalline silicon as background. Different doping concentrations of values: 1 × 10^20^, 3 × 10^20^ and 5 × 10^20^ cm^−3^ for the doped silicon NP are adopted with background of air, which are initially examined to obtain extinction cross sections at the desired MIR range (Fig. 3). It can be seen that at lower doping levels (1 × 10^20^ cm^−3^), the extinction efficiency peaks are at longer wavelengths and with lower intensity owing to the reduced charge carrier concentration, resulting in a decrease in the localized surface plasmon resonance frequency^8^. Since the purpose of this work is to design a MIR absorber, the lower concentration of 1 × 10^20^ cm^−3^ is implemented in all the subsequent simulations. With the silicon background added in the simulations, the resonant wavelength is red-shifted as expected due to the higher refractive index of silicon environment compared to that of air. In addition, the peak is shown to be much broader which is expected to result in the desired broadband absorption in spectral range of interest.

**Figure 4:**
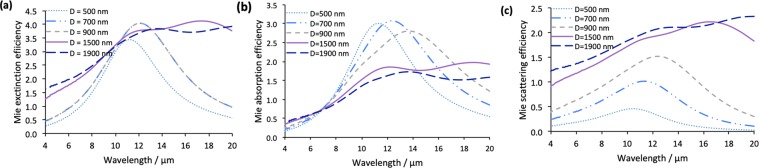
(**a**) Mie extinction efficiency, (**b**) Mie absorption efficiency and (**c**) Mie scattering efficiency of doped silicon NPs with different diameters. A silicon background is implemented in these simulations.

We show that the Mie scattering, absorption and extinction efficiency peaks shift to longer wavelengths as the diameter of the doped silicon NPs increases from 500 to 900 nm as shown in Fig. 4(a–c). It could be seen from Fig. 4(b,c) that the absorption efficiency generally decreases with increasing the NP diameter while the scattering efficiency increases until D = 700 nm. However, at smaller NP diameters (i.e. 500 nm), the absorption starts to decrease again indicating that, below D of 700 nm, the decrease in the filling ratio results in deteriorating the absorption. At diameters larger than 900 nm, the extinction, scattering and absorption efficiency becomes very broad with multiple resonance peaks.

**Figure 5:**
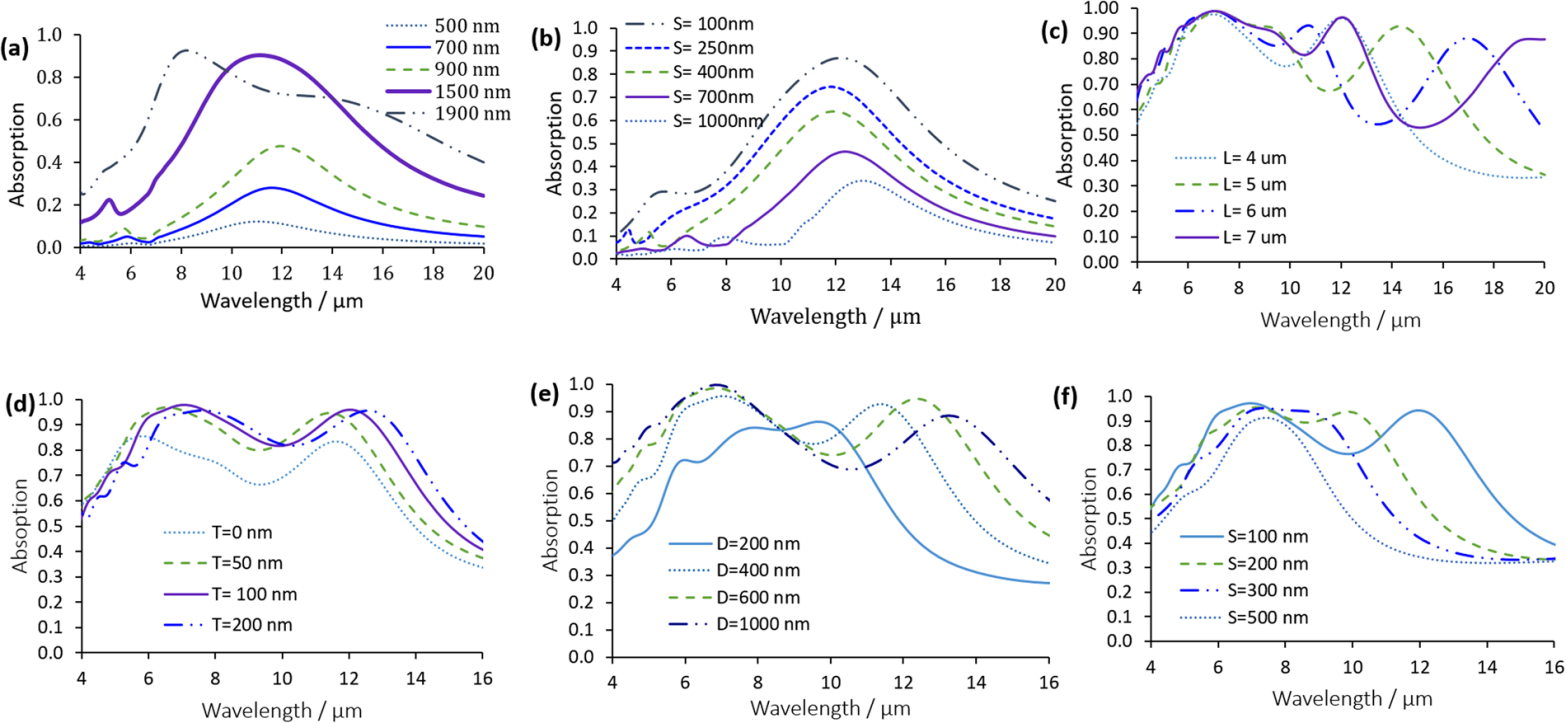
Absorption for (**a**) different NP diameters (the period is constant = 2 µm), (**b**) different NPs spacing (the diameter is constant = 1 µm), (**c**) different NWs length (T = 100 nm, S = 100 nm and D = 500 nm), (**d**) different NW coating thickness (D = 500 nm, S = 100 nm and L = 4 µm), (**e**) different NW diameters (T = 100 nm, S = 100 nm and L = 4 µm) and (**f**) different NWs spacing (T = 100 nm, D = 500 nm and L = 4 µm).

Having observed the above-mentioned resonances within the desired MIR range for 500–1900 nm-sized-particles with doping concentration of 1 × 10^20^ cm^−3^, we proceed to study the absorption of plane MIR waves (λ = 4–20 μm) by the doped silicon NPs embedded in silicon. The simulation region for this design is shown in Fig. 1(c). We find that most of the incident infrared radiation from λ = 9–14 μm is absorbed by the doped silicon NPs with high absorption efficiency of more than 80% at a diameter of 1500 nm. The absorption for different NPs diameters, while fixing the period at 2 µm, are compared in Fig. 5(a) showing that as the diameter of the doped silicon NPs increases, so does the power absorbed. The effect of varying the spacing between the NPs is also studied and illustrated in Fig. 5(b). In these simulations, the diameter was kept constant at 1 µm. It shows that the smaller the spacing (S), the higher the absorption with the highest value obtained at S = 100 nm. Separate absorption in doped silicon and intrinsic silicon for this structure are shown in Fig. S1 in Supplementary Materials.

### N-doped silicon NWs

**Figure 6:**
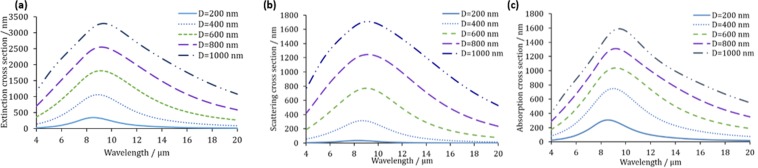
(**a**) Extinction, (**b**) Scattering and (**c**) Absorption cross section of NWs with different diameters. A silicon background is implemented in these simulations.

Figure 6(a) shows the extinction cross section of NWs with diameters ranging from 200 nm to 1 µm. It could be seen that increasing the diameter of the NW results in a significant increase in the amplitude of the extinction cross section, as well as in both scattering and absorption cross sections with a slight increase in the resonance wavelength. The scattering and absorption cross sections are shown in Fig. 6(b,c). The length of the NW is not optimized in this case because this simulation is 2D simulation.

Next, a structure containing doped silicon NWs coated with intrinsic silicon is optimized to obtain the highest absorption in the MIR spectral range. The simulation region of this structure is shown in Fig. 1(b) and it is composed of a 500-nm-thin doped silicon film and a periodic array of doped silicon NWs with different dimensions on top. The doped silicon NWs are coated with a thin layer of intrinsic silicon to increase the surrounding refractive index. Figure 5(c–f) reveals the strong absorption calculated for different coating thicknesses, NWs diameters, spacing between NWs and NWs length. Generally, three main peaks could be observed in all simulations for the different parameters studied. The first two peaks are connected and could be found at λ = 6 and λ = 8 µm for NWs without coating (T = 0). The third peak is located at higher wavelengths (e.g., at λ = 11.5 µm for NWs without coating), as shown in Fig. 5(d). In this figure, different coating thickness are simulated with fixing the other NW paramaters to (D = 500 nm, S = 100 nm and L = 4 µm).

Next, different diameters are simulated with fixing the other NW paramaters (T = 100 nm, S = 100 nm and L = 4 µm). For the structure with T = 0, a broad absorption is obtained from λ = 6 to 12 µm reaching a maximum of 82%. Adding the silicon coating increases the absorption up to a 95%. This is due to trapping the light inside the surrounding silicon, which enhances the overall absorption. This could be confirmed by looking at the separate absorption inside doped silicon and intrinsic silicon coating shown in Fig. S2 in Supplementary Materials. Increasing the thickness of the silicon coating (T) causes a slight increase in the resonance wavelength. For T = 100, 150 and 200 nm, a broadband absorption of more than 80% is achieved from λ = 6 to 14 µm. Peaks of 95% and 93% absorption are obtained at λ = 7.3 µm and λ = 12 µm, respectively, at T = 100 nm.

At smaller diameters of NWs, it could be observed from Fig. 5(e) that the absorption is narrow with the three peaks very close to each other, and a maximum absorption of only 83%. Increasing the diameter is found to increase the amplitude of the resonance peaks up to 99.5% at λ = 7 µm for D = 600 and 1000 nm. Increasing the diameter causes a red-shift in the resonance wavelength of the third peak with slight decrease in absoption after λ = 9 µm. Conversely, the smaller the spacing between the NWs the higher and broader the absorption, and the peaks start to disconnect with the higher wavelength peak shifting to larger wavelengths as the spacing between NWs is reduced. At the smallest spacing of 100 nm, a broadband absorption of more than 70% is achieved from λ = 5 to 13 µm, reaching 95% at 7.2 µm and 93% at 12.3 µm. Increasing the spacing to 200 nm retains the absorption at more than 90% from 7 to 10 µm. The smaller gaps between the NWs result in stronger localized field due to coupling of free electrons across the gap^6^. The different spacing simulations were done with fixing the other NW parameters (T = 100 nm, D = 500 nm and L = 4 µm). In general, it could be noticed that the absorption could be varied in amplitude, broadness and resonance wavelength with different NWs paramaters, in which adjustments could be done according to specific applications.

Finally, the lengths of the NWs are varied from 4 to 7 µm as depicted in Fig. 5(c) while fixing the other parameters (T = 100 nm, S = 100 nm and D = 500 nm). At longer NW lengths, decoupling of the first two peaks starts to appear. A red-shift from λ = 7.5 µm to λ = 12.5 µm is observed when increasing the NW length from 4 to 7 µm. Similarly, the third resonance peak experiences a large redshift from 12.5 to 20 µm as the length is increased to 7 µm. It could also be seen that the absorption reaches 99% for all lengths. For L = 7 µm, more than 80% absorption is achieved from λ = 5 to 13 µm and from λ = 18 to 20 µm.

**Figure 7:**
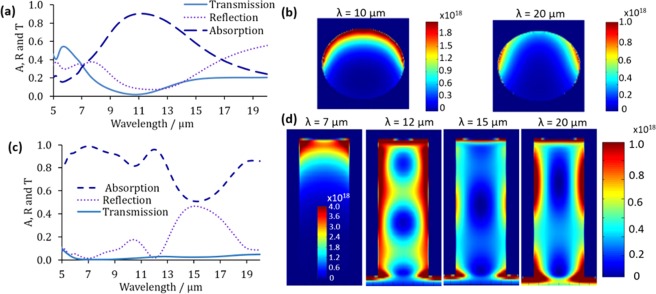
(**a**) Absorption, reflection and transmission spectra and (**b**) absorption profiles at different wavelengths for NPs with 1500 nm diameter. (**c**) Absorption, reflection and transmission spectra and (**d**) absorption profiles of NWs with D = 500 nm, coating thickness = 100 nm, spacing = 100 nm and 7 µm length. The absorption profile represents the absorbed power per unit volume and has the units of (W/m^3^).

The absorption, reflection and transmission graphs of the optimized NPs and NWs structure are plotted in Fig. 7(a,c), respectively, with normal light incidence and TE polarisation. For the 1500 nm-sized particles embedded in a 2-µm-thick film silicon, the absorption reaches 90% at λ centered around 11 µm and is maintained above 50% from λ = 8 to 15 µm. At the highest absorption point (i.e. 10 µm), the absorption profile is shown in Fig. 7(b), where an intense light localization in the upper half of the particle could be observed. At λ = 20 µm, where the absorption declines to slightly less than 50%, the absorption profile shows less light localization around the particles. As for the NWs with D = 500 nm, coating T = 100 nm, S = 100 nm and 7 µm L, a broadband absorption of more than 80% is achieved from 5 to 13 µm. This very high absorption is strongly attributed to the suppressed reflection governed by the unique one dimensional structure of NWs. The better impedance matching between the vaccum and the semiconductor structure resulted in miniziming the reflection and enhancing the absorption. It could also be seen that the transmission is almost negligible. Thus, all the light reaching the NWs is absorbed and the reflection is the main limiting factor of the overall absorption. Therefore, it is believed that the absorption could be further enhanced by having a tapered structure, such as nanocones, to obtain better impedance matching and thus, lower reflection. Another peak could be seen centered at λ = 20 µm. At a lower wavelength, (i.e. 7 µm), the light localizaion is very intense on top of the NW as shown in Fig. 7(d), whereas at longer wavelengths, the light starts to spread0020along the wire. At λ = 12 and λ = 20 µm, light is strongly trapped around the walls of the NWs. However, at λ = 15 µm, where the dip in absorption is found, very faint light localization is observed on the walls of the NWs. This strong localization of light in the NWs significantly enhances the light-matter interaction at the nanoscale in this spectral range, which allows energy harvesting and conversion in MIR. It should be noted here that in all the above simulations, it was assumed that the silicon coating is covering only NWs and not the gaps between them, as shown in the schematic in Fig. 1(b). The actual fabricated devices may contain coating in the gaps between the nanowires to reduce complexity of the fabrication. However, the addition of this silicon coating in the gaps will not affect the simulation results due the very small thickness of the coating. To prove that, the final optimized structure for the NWs is re-simulated with 100 nm silicon coating between the NWs (i.e. in the gaps) and showed no difference between the previously simulated structure (i.e. without coating in the gaps) and the new one. This is shown in Fig. S3 in the Supplementary Materials.

**Figure 8:**
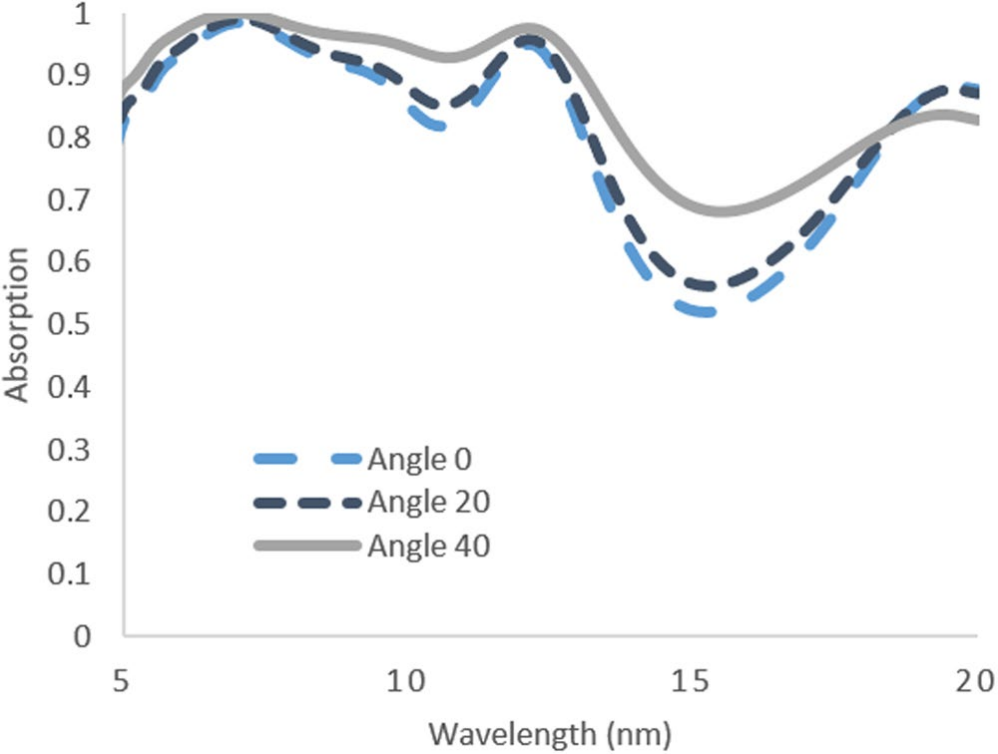
Absorption of optimized SiNWs at different angle of incidences.

Finally, the final optimized structure for the SiNWs is simulated at different angle of incidence to examine the effect of off-normal incidence on the absorption. In these simulations, the periodic boundary conditions are changed to Bloch boundary conditions to account for the phase change and the source is changed to broadband fixed angle source technique (BFAST). The results of these simulations are shown in Fig. 8 and it could be seen that the absorption slightly increased for the source angle at 20^°^. The higher angle (i.e. 40^°^) showed further increase in absorption and this could be attributed to the excitation of additional modes^18,19^.

In conclusion, extremely high broadband absorption in MIR range is accomplished with all-silicon-based structures. Up to 99% absorption is achieved for a structure composed of n-doped silicon NWs coated with intrinsic silicon, and 90% absorption for n-doped Si NPs embedded in intrinsic crystalline silicon. These proposed broadband absorbers are suitable for MIR thermal harvesting applications due to their small footprints, CMOS compatibility and low cost, as well as easy fabrication and integration for on-chip applications. Because these structures could also be easily fabricated with standard silicon fabrication techniques, they are potentially very attractive for commercialization.

## Supplementary information

Supplementary Material

## Data Availability

All data needed to evaluate the conclusions in the paper are present in the paper. Additional data related to this paper may be requested from the author.
