# Supplementary material for: Broadband MIR harvester using silicon nanostructures
Source: Sci Rep. 2019 Apr 9;9:5829. doi: 10.1038/s41598-019-42022-2 (PMC6456598; doi:10.1038/s41598-019-42022-2)
Supplement: Supplementary file 1 — Supplementary Material [file 41598_2019_42022_MOESM1_ESM.docx]

Broadband MIR harvester using silicon nanostructures

**Sara Magdi^1^, Farah El-Diwany^2^, Mohamed Swillam^1,2,a)^**

^1^Department of Physics, School of Sciences and Engineering, American University in Cairo, AUC Avenue New Cairo, 11835 Cairo, Egypt

^2^Nanotechnology Program, School of Sciences and Engineering, American University in Cairo, AUC Avenue New Cairo, 11835 Cairo, Egypt


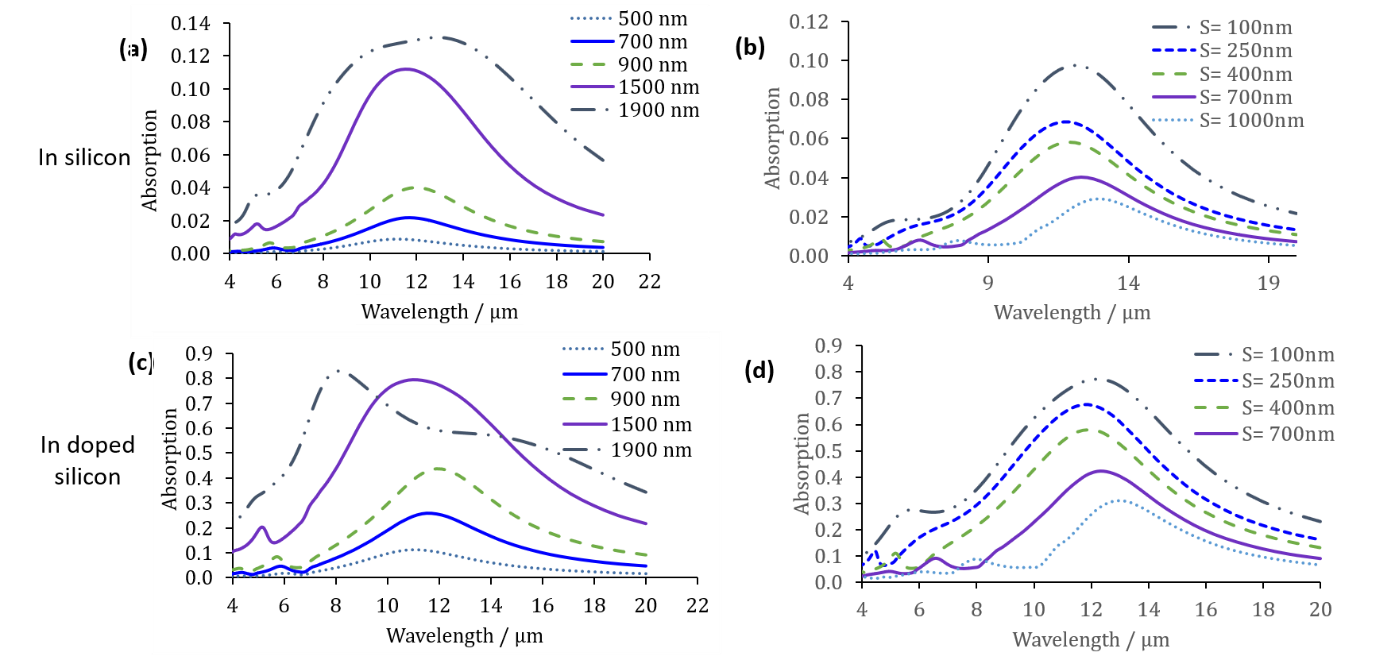


Figure S1: Separate Absorption in Silicon and Doped Silicon NPs for (a and c) different NP diameters and (b and) different NPs spacing.


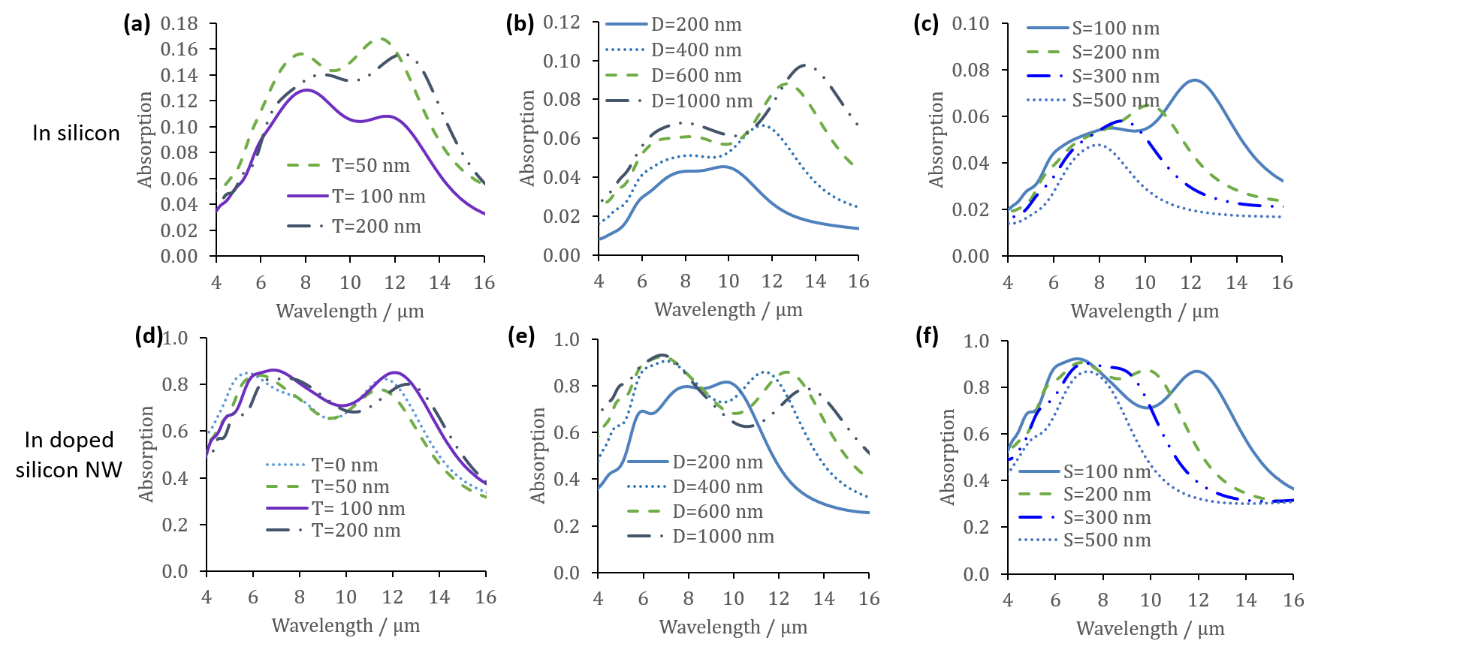


Figure S2: Separate Absorption in Silicon and Doped Silicon NWs for (a and c) different NW coating thickness, (b and e) different NW diameters and (c and f) different NWs spacing.


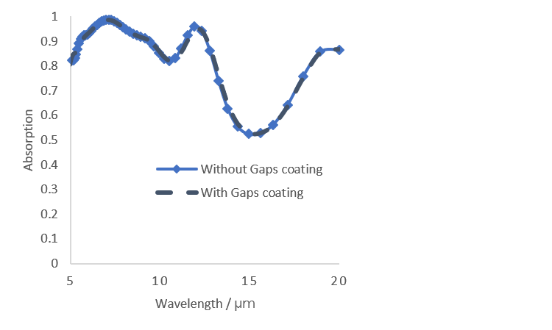


Figure S3: The difference between the simulated structure without silicon coating in the gaps between the NWs and the one with coating in the gaps. It could be seen that there is almost no difference between both results.
